# Address Delivered before the Erie County Medical Society, at Its Annual Session at American Hotel, January 10, 1854

**Published:** 1854-04

**Authors:** P. H. Strong

**Affiliations:** President of the Society


ART. IV.—*Address delivered before the Erie County Medical Society, at its Annual Session at American Hotel, January* 10, 1854. By P. H. STRONG, M. D., President of the Society.

The following address, read before the Erie County Medical Society, seems so well adapted to its purposes and occasion, that the editors of the Journal have requested a copy of it for publication. To this request Dr. Strong has, somewhat reluctantly, consented, leaving upon our not unwilling shoulders the responsibility of subjecting his address to the ordeal of perusal.—EDS.

*Gentlemen of the Medical Society of the County of Erie,*

It seems to be my last official duty to bring to your notice the record of events, incidents and changes pertaining to the condition, and affecting the welfare of our society, that have transpired during my term of office, and to bring up its history to the present moment.

There have been admitted to the society within the year, nine gentlemen, viz., Drs. Merriam, Spearman, Edmonds, Boardman, Ellery P. Smith, Whitney, Jeyte, Gale of Tonawanda, and Joseph R. Smith.

There have removed from our bounds four, viz., Dr. McVean, to parts unknown, though it is believed to California; Dr. Merriam, to Ohio; Dr. Vandeventer, to Texas; and one of our venerated and esteemed senior members, Dr. Wakelee, of Clarence, it is understood, has taken up his permanent residence in Michigan.

Dr. Wallis, formerly of this city, has removed to Aurora, in this county, and continues still in the profession, and one of us.

I have signed officially, certificates to American Medical Association, meeting at New York, for four gentlemen—Drs. G. N. Burwell, White, Congar, and Wilcox, all of whom, it is understood, attended upon its sessions.

I have granted Diplomas, with the seal of the society, after duly attested examination by the proper Board, to two gentlemen: Dr. Edmond E. W. Gale, of Tonawanda, Erie county, Diploma dated June 10, 1853, and to Dr. B. G. Forbush, since settled in Sinclairville, Chautauque county, Diploma dated July 30, 1853.

The year has been one of more than usual healthiness. The summer past has been one, it is thought, of remarkable exemption from epidemic, endemic or contagious diseases. Still the "Reaper," Death, has made inroads upon our ranks, removing two of our number, viz., Dr. Stevens, formerly of Williamsville, (of the exact date of whose death I am not apprised,) and Dr. Joseph Peabody, of this city, who died June 18, 1853, at which event, (as most of you, gentlemen, are aware,) the society was summoned by me to meet to pay the last sad tribute of respect and regard, which was responded to by a large attendance of the society to pass appropriate resolutions, expressive of the sense of the profession at its loss, and soon, thereafter, also, to attend his remains to their earthly resting-place.

Dr. Peabody was regarded as a good exemplification of the high-toned, intelligent, conscientious, Christian physician. He was called from us after some eight years of earnest, patient and honorable effort in our city at establishing himself in a satisfactory practice, at the high-tide of his hopes and aspirations, and the full maturity of his powers.

It may be said with truth, I believe, that for the last eight or ten years we, as a Society and a profession, have had remarkable immunity from death; the more so, that we have passed through, and our mission being to battle with, in close encounter, several fearful destructive Epidemics. It calls for emotions and expression of felicitation and gratitude, Gentlemen, that it has been made so well with us.

One of our number, our respected friend Dr. Sprague, yet remains disabled. His general bodily health has quite improved during the past year—he suffers little or no pain. But his Hemiplegic condition remains unrelieved, and precludes all locomotion and active exercise. His situation, so deeply afflictive, appeals to our profoundest sensibilities and sympathies.

It perhaps is deserving of especial record too, that during my official term the society has "inaugurated an era," and one, as it is regarded, of no trivial significance, by superadding to our annual business gathering, a reunion feature. A dinner, as most are aware, has been provided, both at the last annual and the semi-annual meeting, which, with the accompanying "feast of reason and flow of soul," was so happy in its results, as to suggest the wonder, in the minds of some, (as have other great discoveries) why it had not been thought of before.

Having presented you thus much of the Transactions and the *personnel* of our society, I pass on to some thoughts concerning the present attitude of the profession, which, perhaps, may be characterized as, *Hints at some things which serve as Bars to Progress, in medicine.*

And here, I am aware, Gentlemen, that I have broached a topic upon which the widest variety of sentiment and opinion prevails. It being matter of opinion and judgment, I cannot expect entire assent to my views, nor any assent indeed, that is not the sober deduction of your own observation and reason.

When I speak of obstacles to progress, it may be thought a prerequisite, perhaps, to discussion, to define what I mean by Progress in medicine.

I assume then that the past history of medicine has been, from its earliest dawn to its present state, one of gradual, often slow, but always steady and upward growth—implying, of course, immaturity. Perhaps this position is too obviously true to admit of controversy.

What has been, then, its condition, it is no violence to reason or truth to say, is now; that is as to its character of immaturity. Not to the same degree of imperfectness to be sure—not that great strides toward maturity have not been taken—not that the great names enrolled in the history of its advancement, are not worthy of all honor. I yield to no man in high, reverential estimation of the noble names, "names that were not born to die," that adorn the annals of medicine. Their posterity, especially medical posterity, now on the stage, and for all future time, owes them a debt of honor and of gratitude, which can only be discharged by lifting up and putting on the mantle they have let fall, and going forth in their spirit, and guided by the light they have shed, by their example and by their labors, upon the path, to yet greater achievements. They did much in laying its foundations and beginning its superstructure. They served their generation, and fulfilled their mission, as well as, from the nature of the case, it could be done. But such a science as that of medicine, or rather such a constellation of sciences as surround and converge to the point of forming the art and science of practical medicine and surgery, it is not the work of a generation, nor of many generations, to complete. And if so, we do our predecessors no justice, we pay no true honor to their memories, by coolly appropriating and calmly resting in their labors, and making no effort to push on the car of progress, which has been set in motion, and kept in motion by them, up to our day.

Of what single department of medical science can it be said, we know all that is to be known? I know of none of which it can be said, with even an approximation to truth, but that of general and special anatomy—and here even, the microscopist and the analytical chemist are daily revealing to us that we are but just over the threshold, or at most, a little into the vestibule of the temple of knowledge! Of every other department, how truly may it be said, "there remaineth yet much land to be possessed."

How eminently true is this of the practice of medicine and surgery, the point, perhaps, to which I should limit myself.

Who of us, gentlemen, have not had reason to feel unsatisfied (to say the least) with the results of treatment, at one time or another, in relation to almost every disease of the nosology?

Who of us have not felt, times innumerable, yearnings after some power or agent, more than we have, with which to meet and combat diseased manifestations?

Who of us has not felt in doubt at critical moments, and groped his way in the dark, in reference to location, extent and quality of disease?

Who of us has not experienced, that certain famliar diseases have taken their inception, and stalked through their course, with a kind of "Giant Despair" power, to a fatal culmination, bidding defiance to our utmost exertions for their arrest or abatement? How confessedly are we in the dark as to the causes of disease, whether predisposing, exciting or proximate!

Who has not had occasion, within the few years past, and by the light let in upon the subject by arrant knavery and quackery, to modify his preconceived views of the unaided powers of nature, to cast off the incubus of disease, and by consequence, his views of the powers and effects of certain specific agents upon which, perhaps, he had been wont to rely for such purpose?

In the light of such queries, who can contend for a moment against the sentiment, that "we should count ourselves not to have attained;" or deny that the science and practice of medicine, in nearly every branch or phase of it, is as yet imperfect, and if imperfect, then that it is, and needs must be, progressive?

The truth is, gentlemen, and it cannot be successfully controverted, that our noble and worthy art and science, has ever been, is now, and will be for a long period, if not ever in the future, in a strictly transitional state.

What has been really gained (for we have had, and yet have, much to unlearn that we fondly supposed was real gain) has served, and must serve but as the stepping-stone or ladder-round to somewhat higher.

I know it is more customary to felicitate ourselves, and one another, in thought or word, by what has been accomplished, and what we can and do accomplish every day in our professional walks, for the relief of woes and sufferings.

I said it is customary, and now say, it may be needful and well, on occasions, so to do. But my present purpose is in another direction, and for that purpose, it seems apposite to adopt the sentiment of the poet. "Let the dead past bury its dead," and let us, in the living present believe, that

"Not enjoyment, and not sorrow,
Is our destined end or way,
But to act, that each to-morrow,
Find us farther than to-day!"

If these views are correct, they seem to necessitate the corollaries: that the fundamental and normal law of medicine, as yet, is that of progressive development; and this being conceded, it follows, also, that no one of its practitioners is at liberty to ignore, or to fail to recognize the obligation that rests upon him to do what in him lies for its advancement.

And to this end, while not "forgetting what is behind, we should reach forth to what is before"—laboring with an earnest common purpose, to make all of our experience, observations and reflections, subsidiary to the one worthy end of placing our art and science a step or more in advance of what it was when committed to us. This brings me to the point that I marked out for myself in this discussion, viz: To specify some things in its present state which seem to serve as hindrances to true progress, and helps to the spread of quackery.

It does not fall within the scope of my present purpose to dwell upon those baneful effects traceable to either positively pernicious legislation, or those glaring deficiencies and shortcomings of legislators, by which we are so unrighteously held to rigid responsibilities, and yet denied the only legitimate means to meet them — which ever forces upon one's mind that ancient parallel, the Egyptian taskmaster's demand of "The full tale of brick without straw." Nor, to dwell upon aught that is chargeable to the ignorance, superstition, or cupidity of the nonprofessional public. These, and all other extrinsic hindrances, I leave to other pens and other occasions, and pass on to consider a few things in the profession itself, which, to many minds, serve to defeat its own high end and mission, and to weaken its hold upon public confidence and respect.

I deem no apology necessary before this liberal-minded society for diverging, as is proposed, from the beaten tracks, or for the present free, but I trust, candid expression of sentiments sincerely entertained. It is at once the pride and security of legitimate medicine, that it has that highest style of worthiness, the pursuit of noble ends by noble, openly-avowed means. The profession of medicine can never fall but by its own hands, and whensoever and to what extent soever it fails of the entire confidence of community, it behooves it first, to look well and closely within itself for the cause.

"The fault, dear Brutus, is not
In our stars, but in ourselves (*if*)
We are underlings."

It cannot be denied, and should not be forgotten, Gentlemen, (and its triteness is only relieved by its truth,) that we live in a remarkable age.

Progress is stamped upon everything. Whatever is true of the power and profundity of individual intellects in past ages, it cannot be gainsayed, that there never was an age of so general and intense activity of the human mind, nor an age in which there was so much fearless and unfettered investigation in all departments of human knowledge. There never was an age so fruitful of great results, and in which all previous and all present knowledge is made to bear so directly and effectively upon the well-being of human kind. Never an age of such scrutiny of the claims of past and present experience and observation, to our credence—when the opinions, assumptions, deductions and supposed facts of all time were so mercilessly put to the inquisitorial rack, thumb-screws, and the dross-consuming crucible-fires of searching analysis and inquiry.

And what in this regard is true of knowledge generally, we may not hope our cherished science will escape. Nor does it escape. The most sifting, chaff-scattering tests are, and will continue to be applied to the ground-work and the essential principles of our honored profession, and we must be ready for the ordeal. Now, what constitutes a preparation for it? I answer,

First. A clear recognition of the condition of medicine—that of imperfectness; and of the law of medicine—that of Progress. And it must be regarded as one obstacle to its fulfilling its true mission,—that, so many of the worshipers at her shrine are oblivious to this condition and this law. Not that many, perhaps any, will in words deny that its state is one of incompleteness, nor, that we should try to remedy deficiencies, if it may be effected by tenacious adherence to old views, and old methods and rules of progress and achievement. But many do fail to recognize the obligation that rests upon them, to test new facts and views that are presented to them, even when previous resources and views are confessedly insufficient or erroneous; and many more—the obligation to enter as explorers themselves, and bring to the notice of the profession the results of their explorations. And some there are, it cannot be denied, so steeped in effete conservatism, as to sturdily resist and contest every inch of every step of the progress of a new truth or idea, even when compelled to admit that old supposed facts bearing on the case are unproved, and old views quite insufficient. Now I can conceive that it may be wise in a given case, rather to

> "Bear the ills we have,
> Than fly to others we know not of."

But this must be in departments of knowledge in which the state of actual attainment makes less imperative demands, and in which, also, the means for, and the methods of, true advancement are less clearly indicated than in the science of medicine. In it, enough of underbrush and rubbish has been cleared away to show us where the plow of progress should be driven. Enough is known, at least, to serve as a basis upon which to build still higher. And at no point where we are obliged now to admit onr previous knowledge is imperfect, or our views inadequate, to account satisfactorily for the phenomena that present themselves—in no disease, now regarded as incurable—in no disease (in which we have complete control of our patient, and all his surroundings, and which in spite of our utmost efforts eventuates fatally)—are we at liberty to fold our arms and jump to the presumptuous conclusion that we know all that is knowable; nor to adopt the despairing sentiment, that we have achieved all that is achievable. The truth is, the age is one in which it is neither safe nor wise to admit to ourselves, or contend before others, that we have reached the utmost limit of achievement, in aught wherein our present knowledge is in the least imperfect, or our resources for the removal of disease, in the least inefficient.

A living author (of our own profession) says most truly: "The current of knowledge and improvement rushes on so strongly, that they who hesitate to commit themselves to it, will soon be left far behind, and serve only the disgraceful purpose of enabling us to measure the force and rapidity of the stream."

True conservatism is well. "Conservatism in progress" I regard as a fitting motto to inscribe upon the banner of legitimate medicine. But that absurd conservatism, which, while it is obliged to confess to great imperfectness of actual resources in certain cases, and yet frowns upon all innovation, I have the least possible respect for. To be measurably in doubt as to existing, and yet vailed phenomena, or, as to appropriate resources in a given case, is, in the present state of medicine, frequently a necessity. To always admit it to ourselves, and on fitting occasions to others, is candid and worthy of commendation. But to stand by, coolly oblivious to the obligation that rests upon us to clear up the doubt, and advance any step that is possible; or, to fail to hail with joyful welcome any light that may perchance be reflected upon the point by other observers; or, to treat with contemptuous indifference the researches and results of those who are inclined and competent to enter fields of investigation for which we may lack the taste or ability; or, worse still, to meet with disingenuous criticism and obloquy, the well-directed or well-intentioned efforts of such, is a bogus conservatism that makes a truth-loving, earnest mind, in or out of the profession, ache to fly in the face of, whensoever it is manifested.

Regret it as we may, to just the extent that this is shown, we shall fail to secure the honor and the confidence of a discriminating public, and what is more and worse, we shall deserve to fail.

The true attitude of our profession then seems to be, while we hold with firm grasp to the truth which has been elicited, whether practical or speculative, we should reach forth toward what is undeveloped, or unsettled, and learn by our own successes and failures; learn by all the light that is reflected from the observations and experience of our "brethren in arms;" learn by the light unwittingly shed by the blundering good or ill-luck of reckless quackery; learn by every and all means, that can be made subservient to the advancement of our science and art.

We may well glory in the history of medicine; we may well exult in what has been achieved, and in the state and amount of our resources for the relief of suffering humanity; but, to my mind, the strength and impregnability of honest medicine has ever been, and will ever be, in that, while its purposes and aims are pure and noble, it ingenuously pursues those ends by means not less worthy; eliciting and developing gems of truth from every placer and quarry, and appropriating and reflecting it, whencesoever obtained, as capital joint-stock, common and open to all, and upon which all may draw and levy in the contest we wage with disease and death. This it is, I humbly conceive, that places regular medicine at a world-wide remove from every phase and form of partialism that has ever lifted its puny arm against it, or that has ever infested and afflicted society.

This it is which constitutes the Gibraltar of medicine, against which all the waves of ignorance, and all the billows of knavery, may dash, and fret, and spend themselves, and serve only to prove their own foundation to be in the sand, and ours upon the rock.

Bar to Progress No. Two. Worthy of being thus characterized as it seems to me, is the habit of so many in our ranks to ignore the claims that medicine, objectively considered, has upon them to labor for its advancement.

Pursued and depended upon as a means of maintenance, as it is, and many of us being necessitated to struggle earnestly to secure such maintenance for ourselves and those dependent upon us, it is not unnatural, perhaps, that our thoughts and efforts should be so largely directed to, and bounded by, this phase of obligation, and that other superadded, and yet imperative one, our duties to our patients. (I speak not in this connection of our duties to our God, the necessity being precluded by my view, that all our duties should be done as to Him, and being the greater, includes the less.) I shall merely refer to, and contend for, in this discussion, the third phase of obligation, our duty to our profession, considered both as a science and art, and as represented by its promoters and practitioners.

I think it can be but regarded as a statement of a fact, that very many in the profession are, (from the above and somewhat, perhaps, extenuating circumstance,) or from other causes, habitually forgetful or neglectful of this obligation. From whatever cause, however, and to just the extent it exists, it is equally a fact, that it is no trifling obstacle to progress. How has medicine been promoted in the past, and how came we possessed of the rich legacy that is handed down to us in the vast treasures of knowledge and facts accumulated for our use and the use of all time? How otherwise, than by the individual efforts, the patient, untiring, and unselfish labors of those who have gone before us. The growth of medicine has been for the most part neither spontaneous nor accidental. The history of medicine reveals no May-day game — "but a Battle and a March." It has only advanced, and from the nature of the case it can only advance, by the patient and laborious investigations, observations and deductions of those devoted to it; and to toil for this end is an obligation that rests upon one no more than another. It rests alike upon all, and is only to be limited by the ability and opportunity of each and every individual engaged in it. It follows, we are not only bound to apply our personal experience and knowledge to our individual benefit, and that of our patients. But in a sense a higher (because more unselfish) duty devolves on us, to bring all our gains, of knowledge and of facts, to the notice of the profession, and deposit them as our contribution to the common stock, upon which medicine depends for its honor, and its means of usefulness.

In this view, no physician "liveth to himself" — no one liveth to his patients, merely. But each must feel the obligation as imperative, to do all that in him lies, to lessen the area of the *terra incognita*, the hitherto uncultivated or unexplored fields, that lie all around us; and to root out the pernicious seeds of error, whether of doctrine or of practice; and to lay up positive durable treasures in the archives of medicine, from which it may be said of us when we are laid aside, not only that we achieved honor, respectability, and a competency for ourselves; not only that we served our employers faithfully and well; but also, that medicine is some step or steps in advance, and therefore the better, in that we have lived.

I know not but it may be deemed an extreme opinion, but it is one fully entertained, that constituted as medicine is, no one is at liberty to appropriate to himself and make himself the exclusive depositary of any knowledge of fact, or of interpretation of fact. No one may know any thing (which he regards as new and as important, and which is not as yet apprehended by others) that he is not willing, nay, desirous of contributing to the common stock. All this seems incontrovertible, and it may be asked by what means it is proposed to effectuate so much of correct sentiment? At the present age, and in the present state of the profession, the two great instrumentalities for the advancement of medicine, viewed objectively, are the Press and Association.

I propose not to dwell upon the importance nor the appropriate functions of either. But I shall content myself to insisting, that by one means or the other, and by both when opportunity presents, we are bound by the very constitution of medicine, and the very nature of our relations to it, to labor earnestly, and with a will, for its promotion. This obligation then is neither incidental, nor optional, but inherent and imperative, and can no more be shaken off than our duties to our patients and to ourselves. If charity should "begin at home," we are nowhere told it should end there. But it is to begin there, in the present case, for the very purpose that it may end elsewhere.

The power and advantages of the serial press is a fruitful theme that I propose not to dwell upon, but it may be safely said, methinks, in no department of human thought and effort, has it achieved greater victories, or more enduring benefits, than in the province of medicine.

The necessity of receiving and absorbing light therefrom, is, perhaps, more generally recognized than the duty of radiating and reflecting light through the same medium; and yet the one privilege (of enjoying and profiting by the labors of others) inasmuch as no one is more responsible than another, implies a general and individual obligation of communicating, for which all have the competency, and also the facilities, to some degree.

The advantages of organized stated associations of medical men for individual and combined effort and improvement, are not less apparent. So far as pertalns to its function of eliciting, developing and diffusing truth, it needs not now to be dwelt upon. But it has an additional, and, perhaps, not less important function — that of cultivating and perpetuating the amenities of professional life.

It is matter of common observation, that in the absence of this means, the points of genial contact between medical men, are too few and intangible for harmonious, cordial fellowship. Partly from causes inherent in general society; partly from the defective perception of their duties by many of the profession; and in part also from the very nature of our vocation, with other causes; suspicions, alienations, and animosities are apt to be engendered, which are certain to grow and thrive, and attain, it may be, monstrous proportions, while men are kept at arm's length; which are nearly as certain to dwindle into pigmies, or vanish into "airy nothings," when the frowning parties are brought together in social relations, and in the spirit of true manhood, assay to understand each other. Many a mountain of ill-feeling has dwindled into a mole-hill by a close survey of each other's views and positions, and that too without the abatement of a single iota of real dignity, but its wonderful enhancement—illustrating for the thousandth time that "'T is distance lends (if not enchantment) at least importance to the view." To fulfill these high and urgent indications, association for mutual and unselfish ends is most happily adapted, and what is more it is nearly all in the present order of things in our profession, that meets this great want so often indicated. Hence it is, that an obligation rests upon all that can neither be denied, nor should be ignored, to sustain with interest, and to feel responsible for the character, of such organizations. The failure, then, to respond to the claims that medicine has upon us to communicate as well as to receive, so widely prevalent, it must be admitted to the full extent that it exists is no slight Bar to Progress.

The third, and last, to which reference will be made at this time, is to be found in the feuds and bitterness of feeling, existing, too often, among medical men.

Strange, passing strange, is it, that in a calling whose ends are so lofty, and honorable, and merciful, and the daily practice of which is so full of genial, humanizing influences—in which every day's duties testify to appeal to our tenderest sensibilities—that notwithstanding all this, it should be pre-eminently obnoxious to the charge, that its votaries indulge in the intensest, most inextinguishable animosities. This is said of us so generally, as to be well nigh proverbial. And for "doctors to disagree," and that, too, bitterly, is thought to be, almost a matter of course, an inherent necessity.

It may be true that from the nature of things, occasions more frequently arise with us for differences of opinion and judgment than with any other of the learned professions.

It is true, doubtless, that in no other arises such frequent necessity for an honest and candid expression of such differences as exist. It is especially true, from the trying and fearful responsibilities often cast upon us, that nowhere is there so imperative a demand for prompt consideration, and independent action upon our own opinions and judgment, as with us; and that hence, any impertinent interference or officious tampering with either our rights, feelings, or reputation, by unsettling the minds of our patients or their friends, and thus defeating our cherished intentions, and, perhaps, our almost engrasped hopes, constitutes a kind of *experimentum crucis*, or torturing test to one's patience, to which few or no other callings in life, are either exposed to, or can appreciate. But is it true that all this furnishes any justification for allowing ourselves to be despoiled of our peace of mind? Is it true, that we may for all this turn our hearts into fountains, from which the bitter waters of strife and anathema shall perennially flow?

Can this, or any thing, serve to extenuate that ferocity of hatred too often seen, when a professional brother cannot be spoken of, hardly thought of, without curses, deep and loud; more than this, can anything serve as an apology for denying the professional rights, by refusing the professional courtesies to one with whom we stand in professonal covenant?

For dereliction in duty worthy to excite remark, or feeling, we have appointed appropriate tribunals for redress, and are morally bound to abide by them. If we have a case worthy to excite indignation, and from which we have righteous cause for feeling aggrieved, it promotes neither our personal interest, nor dignity, to be unwilling to submit it to those tribunals. Nay, it is a confession to the poverty and weakness of our cause, and hence, an act of self-stultification.

Have we any right to take the means of redress into our own hands by openly condemning and refusing professional courtesies to any one whom we have not presented for trial at the constituted tribunal? If so, the bonds which hold us together as a profession, are a mere rope of sand. And to take to ourselves the dignified appellation of an honorable, liberal, high-minded profession, is a mere flourish of rhetoric, which can only entitle us to be credited for "sounding brass or tinkling cymbal." Look at the habit in this regard, of any other profession—for instance, the legal. Is it ever seen that personal jars, and bickerings, are allowed to be magnified into such overshadowing importance that its practitioners cannot observe professional civilities in the discharge of professional duties—or, that they will not meet on some common ground, when occasion or the demands of the profession requires? Rarely, if ever.

The truth seems to be, too often with us, that our personal piques and dislikes are nurtured and tended, and allowed to assume an undue, sometimes monstrous, importance and perpetuity, which is out of all proportion to the alledged causes, and indulged with utter indifference to the character and claims of the profession.

To agree like men is a good thing. To differ, like men, is nobler, and better. To agree when we can, and to agree to differ, when we must, and yet to curb differences, alienations even, from degenerating into spite and rancor, is an obligation from which no sophistry, no imaginary nor real grievance, can relieve us, while we continue to stand upon a common platform, and labor for a common end.

The causes for the unnatural and unnecessary prominence of our jars and jostlings, are manifold — neither my time, nor your already overtaxed patience, will allow even of allusion to them all — allusion will therefore be restricted to one or two of the more prominent.

First — the innate love of war and contention which characterizes not a few in the medical world. There have ever been found minds so mal-attuned that the sweetest music that greets their ears through life, is the shout and the shock of battle — the din and confusion of conflict. Peace, and the pursuits which can only thrive in, and the advantages that can only flow from it, are just nowhere in their regard. To the behests of medicine on her own account, and to the pleadings of community on its own account, for the sheathing of the sword, to each alike a deaf ear is turned. War is their normal condition, their very *pabulum vitæ*, and sometimes never ceases while they tread the earth.

Again: ambition of leadership and desire for personal aggrandizement, is regarded as not unfrequently a cause. Under the guise of "regard for the honor of the profession," there often seems to lurk, sometimes intentionally, sometimes unconsciously, a design, or desire, to compass some selfish end; some personal "axes are to be ground," which requires the tightening of the belts, and the letting on of the "waters of strife." The forces are marshaled; the battle is set in array; the roll is called; and, whoso in his honest simplicity ventures to question either the righteousness of the cause, or the legitimacy of its leader, is looked upon as no better than he should be, or treated as an open enemy.

Now, I say, away with the cringing servility that cowers before such intolerant demands! And scouted be the domineering insolence which dictates them, come they from any one man, or any set of men!

If men in our ranks are determined not to pursue "the things that make for peace," but will bend all their energies to kindle and fan the flames of interminable discord and war, whether from their innate relish for it, or to promote their own selfish projects, let the profession rigidly allow them to grind their own axes, and fight their own battles!

Eschewing the partizan spirit, and scorning leadership dictation, let us weigh every question by its own merits, and judge every man by his intrinsic qualities.

Soaring above the scenes of conflict, let us attain that serene height, from which we may clearly perceive, and in which we may calmly, yet earnestly, labor for the true honor, the real welfare, the peaceful advancement, of our truly noble art and vocation.

It is more than mortal's truth, that "A house divided against itself, cannot stand," and I know not what can relieve a profession, which, by its own ever-warring elements, has reached essentially the same condition, from sharing the same fate.

It is matter of observation, of the truth of which I am fully persuaded, that certain popular phases of quackery unsettle most the public mind in, receive the greatest impetus, and derive the most substantial aid and comfort from, those communities, in which our profession is the most inharmonious and jarring. If it is so, is it not a fact of significant import? But, be this as it may, it is matter of profound conviction with me, that, with annals so inspiring and glorious, as are ours, and with a living profession, by every man of which its true mission is fully pondered; its condition and law clearly perceived; its claims and obligations cheerfully and honestly responded to; presenting to the world an undivided front, as a band of brothers, each one in his sphere regarding the reputation and the rights of every other one dear as the apple of his eye—so constituted, and so fulfilling its mission, it would be a power, in our country at least, which, for securing any legitimate end, would be well-nigh omnipotent. Before it, every species and form of quackery would vanish, like the early dew before a summer-sun.

Like the well-constructed arch, with which, the greater the pressure brought upon it, the firmer it becomes; so legitimate medicine would be seen to be stronger, in itself and in the world's esteem, by every fresh test of her strength in the responsibilities imposed upon it.

In conclusion, Gentlemen, I thank you for the honor conferred upon me by calling me to preside over your society. I thank you for the kindly forbearance manifested throughout my very imperfect discharge of its duties, and especially for the patience with which you have borne this last infliction of what, I fear, may be regarded as crude and ill-judged sentiment. I can only farther say, Gentlemen, I wish you, both individually and as a society, a long career of merited prosperity and happiness.

